# Overexpression of snapdragon *Delila* (*Del*) gene in tobacco enhances anthocyanin accumulation and abiotic stress tolerance

**DOI:** 10.1186/s12870-017-1015-5

**Published:** 2017-03-23

**Authors:** Aung Htay Naing, Kyeung Il Park, Trinh Ngoc Ai, Mi Young Chung, Jeung Sul Han, Young-Wha Kang, Ki Byung Lim, Chang Kil Kim

**Affiliations:** 1 Department of Horticultural Science, Kyungpook National University, Daegu, 4165122 South Korea; 2 Department of Horticulture & Life Science, Yeungnam University, Gyeongsan, 712-749 South Korea; 3 Department of Agricultural Education, Sunchon National University, Suncheon, South Korea

**Keywords:** Antioxidant activity, bHLH protein, Drought stress, Salt stress, Transgenic tobacco

## Abstract

**Background:**

Rosea1 (*Ros1*) and Delila (*Del*) co-expression controls anthocyanin accumulation in snapdragon flowers, while their overexpression in tomato strongly induces anthocyanin accumulation. However, little data exist on how *Del* expression alone influences anthocyanin accumulation.

**Results:**

In tobacco (*Nicotiana tabacum* ‘Xanthi’), *Del* expression enhanced leaf and flower anthocyanin production through regulating *NtCHS*, *NtCHI*, *NtF3H*, *NtDFR*, and *NtANS* transcript levels. Transgenic lines displayed different anthocyanin colors (e.g., pale red: T_0_-P, red: T_0_-R, and strong red: T_0_-S), resulting from varying levels of biosynthetic gene transcripts. Under salt stress, the T_2_ generation had higher total polyphenol content, radical (DPPH, ABTS) scavenging activities, antioxidant-related gene expression, as well as overall greater salt and drought tolerance than wild type (WT).

**Conclusion:**

We propose that *Del* overexpression elevates transcript levels of anthocyanin biosynthetic and antioxidant-related genes, leading to enhanced anthocyanin production and antioxidant activity. The resultant increase of anthocyanin and antioxidant activity improves abiotic stress tolerance.

## Background

Anthocyanins are plant pigments produced via a dedicated biosynthetic pathway in flowers, fruit, leaves, and stems. Ornamental plants with novel colors and coloration patterns are of great commercial value in the floricultural industry, as they are among the most important aesthetic characters for consumers. Plant breeders have developed various flower colors using natural mutants or interspecific hybridization. However, current breeding techniques cannot create particular flower colors in some plant species due to hybridization barriers. Genetic engineering offers an alternative approach that has successfully developed these desired flower colors in common floricultural plants, including roses and carnations [1].

The transgenic expression of anthocyanin regulatory transcription factors from ornamental plants led to anthocyanin production in other plants [1]. For example, anthocyanin production was enhanced in tomato with the co-expression of snapdragon (*Antirrhinum majus*)-derived bHLH transcription factor *Delila* (*Del*) and the MYB transcription factor *Rosea1* (*Ros1*) [2, 3]. Furthermore, *AtMYB12*, *Del*, and *Ros1* overexpression enhanced anthocyanin biosynthetic gene expression, as well as activating primary metabolism [4]. However, only a single study to date has examined the effect of of *Del* alone on anthocyanin production (in tobacco) [5]. Thus, more data are necessary to fully understand of the involvement of this gene in anthocyanin-linked flower pigmentation.

Ongoing global warming scenarios in climate models have predicted that drought severity and soil salinity will steadily increase (IPCC 2007). Both are major abiotic stressors; drought severely arrests plant growth and development through a reduction in cell division and expansion rates, leaf size, stem elongation, root proliferation, and water use efficiency [6]. These changes eventually cause crop biomass a reduction. Similarly, salinity alters many physiological and biochemical processes that disturb normal plant growth and development [7], thus adversely affecting crop productivity and quality [8]. Abiotic stress can increase reactive oxygen species (ROS), leading to cell damage from oxidative stress [9]. Plants cope with the damage through generating antioxidant enzymes, such as superoxide dismutase (SOD), catalase (CAT), peroxidase (POD), and ascorbate peroxidase (APX).

Previous studies have reported that plants with high anthocyanin content also possess high antioxidant capacity. Moreover, these plants exhibit resistance against a variety of abiotic and biotic stressors, including salinity and drought, as well as microbial and fungal attacks [10, 11]. This increased general tolerance is attributable to antioxidants scavenging ROS that impede plant growth (e.g., hydrogen peroxide, singlet oxygen, superoxide radicals [12, 13]. Thus, in addition to its role in plant coloration, anthocyanins are important antioxidants responsible for protection from major stressors. In support of this function, a recent study suggested that high anthocyanin content in transgenic tobacco enables the plants to tolerate relatively low ROS accumulation under chilling stress [14]. Similarly, antioxidants were shown to be important in protecting plants against salt-stress-induced oxidative damage [15]. Furthermore, flavonols are compounds with high antioxidant activity, and the upregulation of their biosynthesis increased protection against UV radiation in *Ligustrum vulgare* plants [16]. Potato plants overexpressing transcription factor *IbMYB1* exhibited enhanced anthocyanin production and improved salinity tolerance [17]. Anthocyanin biosynthetic genes *PAL* and *CHS* are proposed as candidates for molecular breeding to increase crop resistance to wounding and salt stress [18]. Finally, Fini et al. [19] and Nakabayashi et al. [20] showed that improved abiotic stress tolerance under enhanced flavonoid accumulation is linked to greater ROS scavenging ability. However, these previous works did not directly investigate the association between plant anthocyanin content and abiotic stress tolerance. Hence, our research aims to address this gap.

In this study, we generated transgenic tobacco expressing snapdragon-derived *Del*, and then investigated its role in anthocyanin production using phenotypic and molecular characterization. In addition, we examined stress-induced antioxidant activity and related gene expression to understand how increased anthocyanin content from *Del* overexpression affected salt and drought tolerance.

## Results

**Fig. 1:**
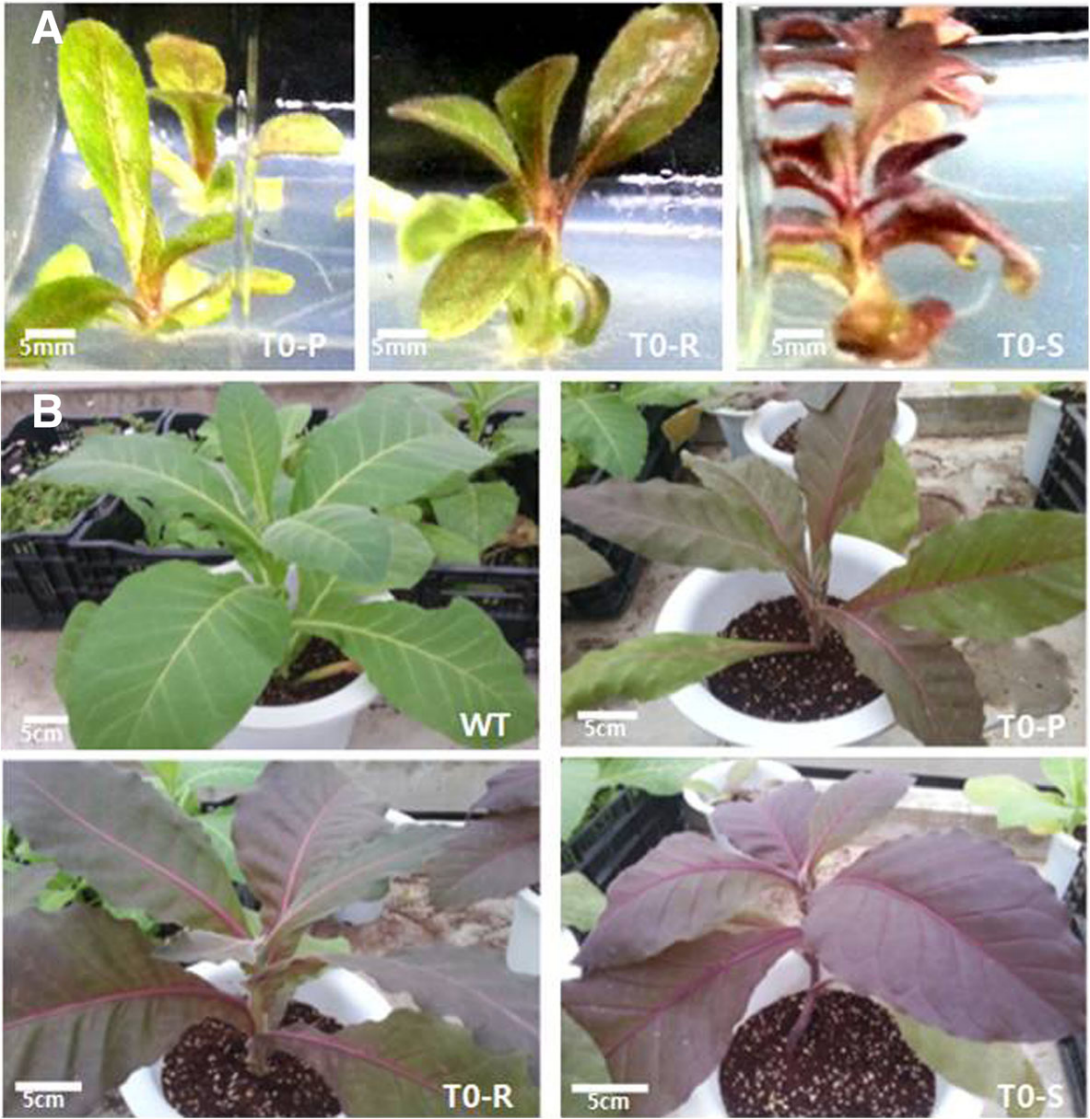
Comparing anthocyanin-content phenotypes across (**a**) three independent *Del*-overexpressing transgenic lines (in vitro stage) and across (**b**) WT and transgenic lines (in greenhouse conditions). All lines exhibited different phenotypes. T_0_-P, T_0_-R, and T_0_-S refer to pale red, red, and strong red transgenic plants in the T_0_ generation, respectively

The T_0_ transgenic phenotypes displayed stem and leaf colors corresponding to differing anthocyanin accumulation levels: pale red (T_0_-P), red (T_0_-R), and strong red (T_0_-S) (Fig. 1a). Upon transferal to a greenhouse alongside wild type (WT), anthocyanin accumulation was maintained in transgenic lines and obvious between-line phenotypic variation was observed (Fig. 1b).

Homozygous lines in the T_2_ generation were verified using Mendel’s segregation laws (data not shown). Next, T_2_-P, T_2_-R, and T_2_-S plants were selected for analysis of anthocyanin content and molecular characterization.

**Fig. 2:**
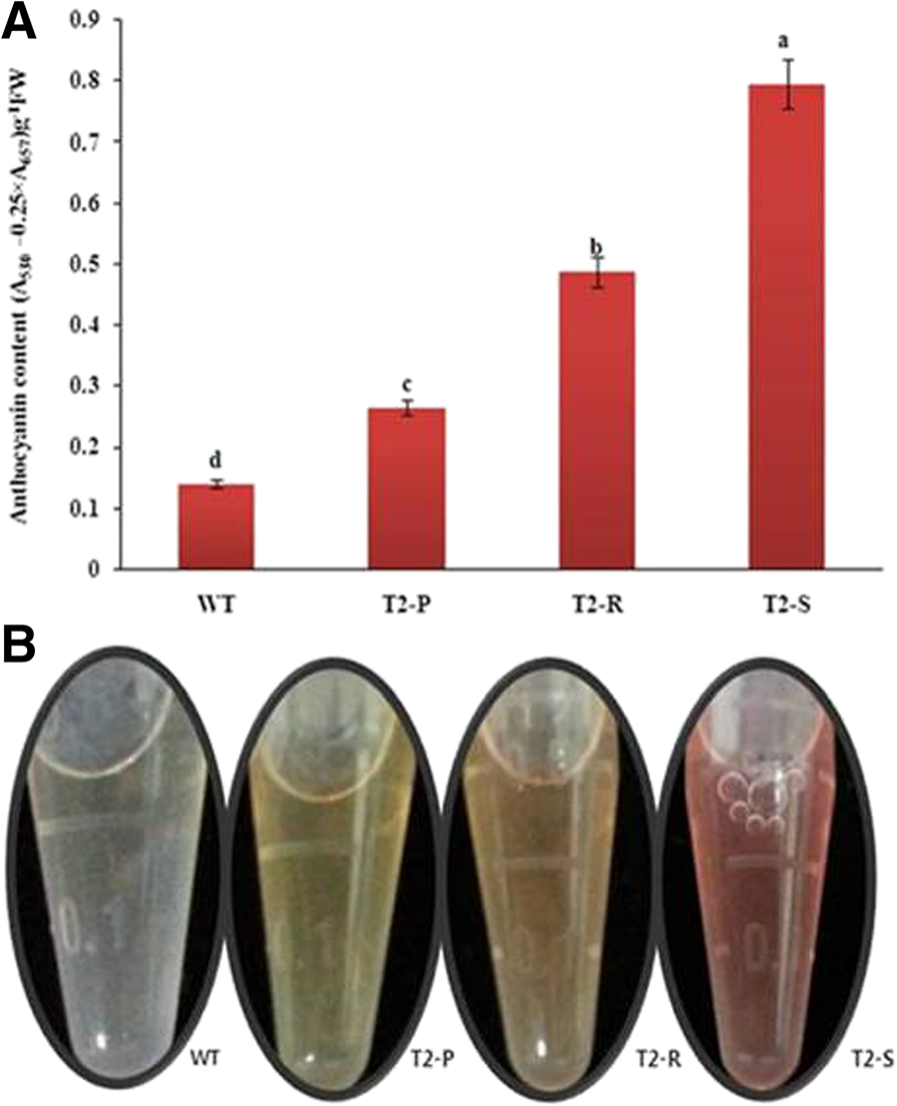
Comparative analysis of (**a**) anthocyanin content and (**b**) anthocyanin extract color in WT and transgenic *Del*-overexpressing plants T_2_-P, T_2_-R, and T_2_-S. Error bars indicate the standard error (SE) of average anthocyanin content

Leaf anthocyanin content was highest in T_2_-S plants, followed by T_2_-R, T_2_-P, and finally WT (Fig. 2a). Anthocyanin content was also associated with the color of each extract (Fig. 2b).

**Fig. 3:**
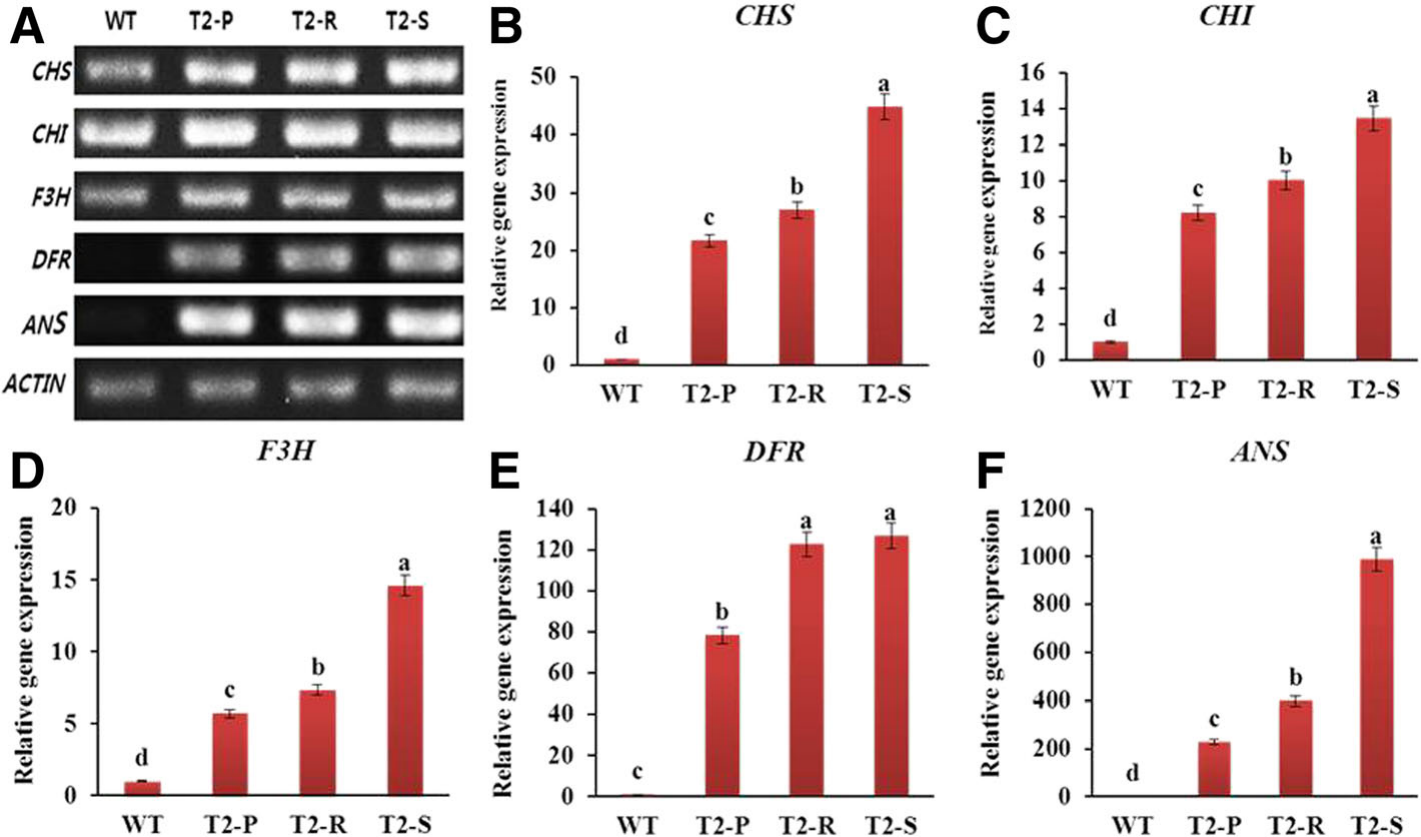
(**a**) RT-PCR analysis of gene expression and (**b**-**f**) qRT-PCR analysis of anthocyanin biosynthetic gene transcripts in leaves from WT and transgenic *Del*-overexpressing plants (T_2_-P, T_2_-R, and T_2_-S). Error bars indicate the standard errors (SE) of average results

The results of RT-PCR indicated that all investigated anthocyanin biosynthetic genes (*NtCHS*, *NtCHI*, *NtF3H*, *NtDFR*, and *NtANS*) were expressed in the leaves of transgenic lines, while *NtDFR* and *NtANS* were not expressed in WT leaves (Fig. 3a). Thus, *Del* overexpression appears to regulate all major genes involved in the anthocyanin biosynthetic pathway. Moreover, low anthocyanin content in WT corresponds to the lack of *NtDFR* and *NtANS* expression.

We further investigated gene transcript levels using qRT-PCR to understand why anthocyanin accumulation differed across transgenic lines, even though they all expressed anthocyanin biosynthetic genes post-*Del* overexpression. Gene expression in the transgenic lines was correlated with anthocyanin accumulation: the highest transcript levels were found in T_2_-S, followed by T_2_-R, T_2_-P, and finally WT (Fig. 3b-f). Notably, *DFR* and *ANS* expression was significantly higher in T_2_-R than in T_2_-P. Additionally, T_2_-S and T_2_-R had similar DFR transcript levels, but the former exhibited significantly higher expression for all other genes.

**Fig. 4:**
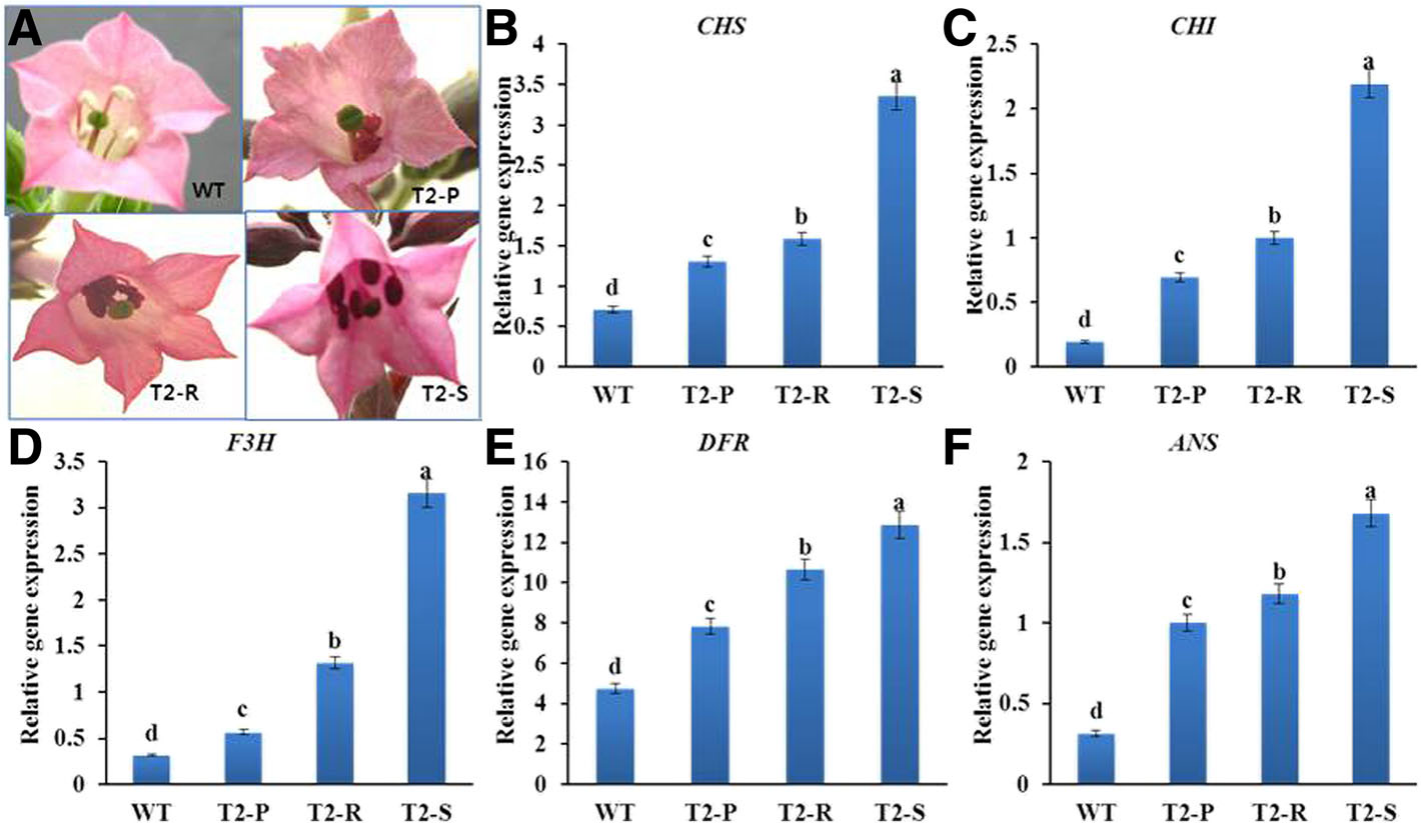
Comparison of differences in flower phenotype (**a**) and (**b**-**f**) qRT-PCR analysis of anthocyanin biosynthetic gene transcripts in flowers from WT and transgenic *Del*-overexpressing plants (T_2_-P, T_2_-R, and T_2_-S). Error bars indicate the standard errors (SE) of average results

Transgenic flowers were redder than the pale pink of WT, ranging from dark pink (T_2_-S), orange pink (T_2_-R), to pink (T_2_-P) (Fig. 4a). We next used RT-PCR to examine how flower color is influenced by genes controlling the anthocyanin biosynthetic pathway (*NtCHS*, *NtCHI*, *NtF3H*, *NtDFR*, and *NtANS*). All analyzed genes were expressed in both transgenic and WT flowers, differing from leaf tissue expression patterns with no WT *NtDFR* and *NtANS* expression. However, as observed for leaves, transcript levels of all biosynthetic genes were highest in T_2_-S flowers, followed by T_2_-R, T_2_-P, and lastly, WT flowers (Fig. 4b-f).

### Environmental stress experiments

#### Antioxidant activity in transgenic lines and WT before stress treatment

**Fig. 5:**
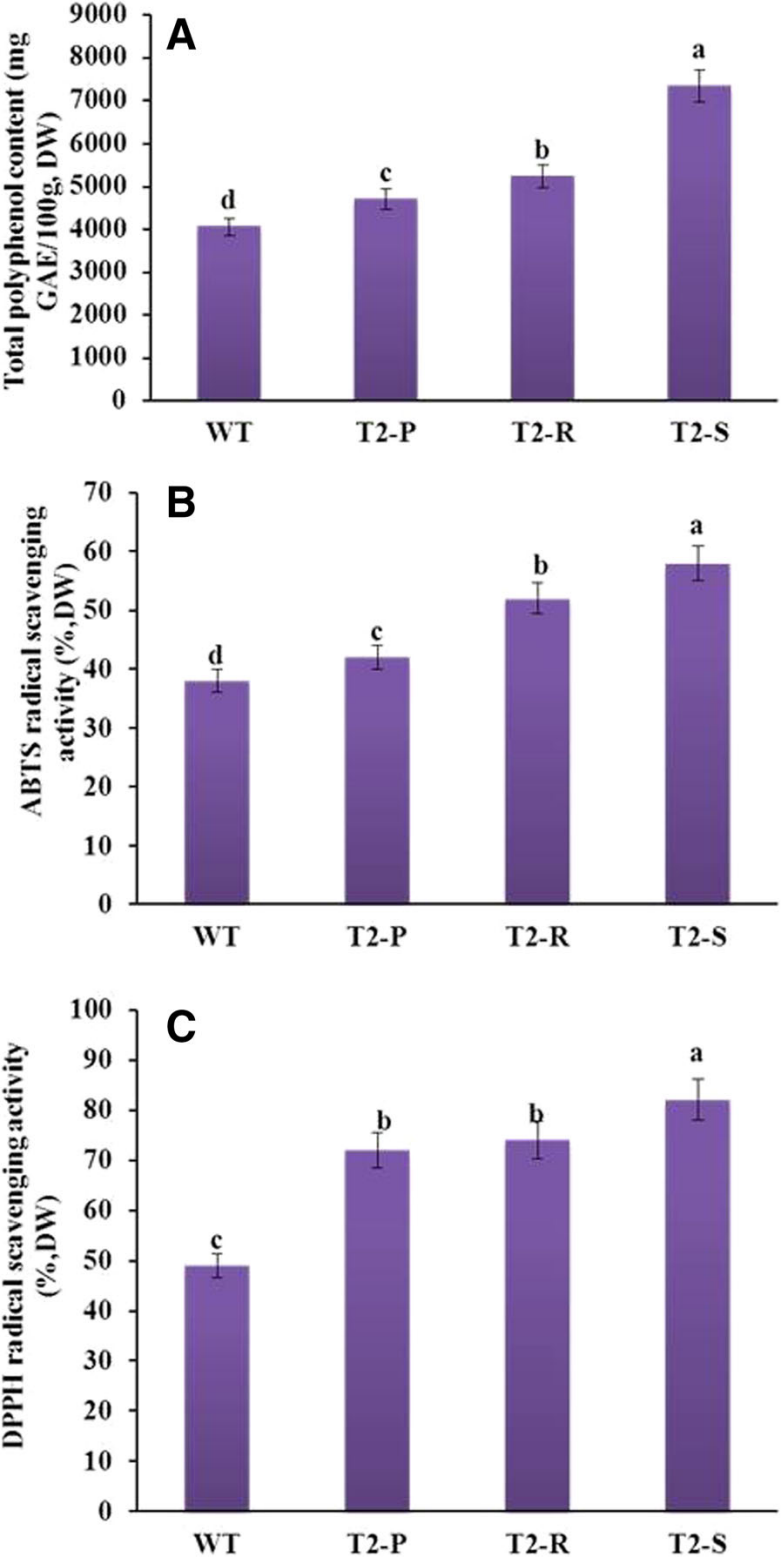
Antioxidant activities under normal growth conditions. (**a**) Total polyphenolic content, (**b**) ABTS radical scavenging activity, and (**c**) DPPH radical scavenging activity. Data from WT and transgenic *Del*-overexpressing plants (T_2_-P, T_2_-R, and T_2_-S) are shown. Error bars indicate the standard errors (SE) of average results

To evaluate the association between anthocyanin content and antioxidant activity, we analyzed total radical-scavenging activity (2,20-azinobis [3-ethylbenzothiazoline-6-sulfonic acid] diammonium salt: ABTS and 1,1-diphenyl-2-picrylhydrazyl: DPPH) and total polyphenol content of leaf extracts from non-treated four-week-old T_2_-P, T_2_-R, T_2_-S, and WT plants. Antioxidant activity and total polyphenol content were highest in T_2_-S and lowest in WT, with T_2_-R and T_2_-P in between (Fig. 5). This pattern indicated that higher anthocyanin content in transgenic lines leads to higher antioxidant activity.

### Salt stress tolerance

**Fig. 6:**
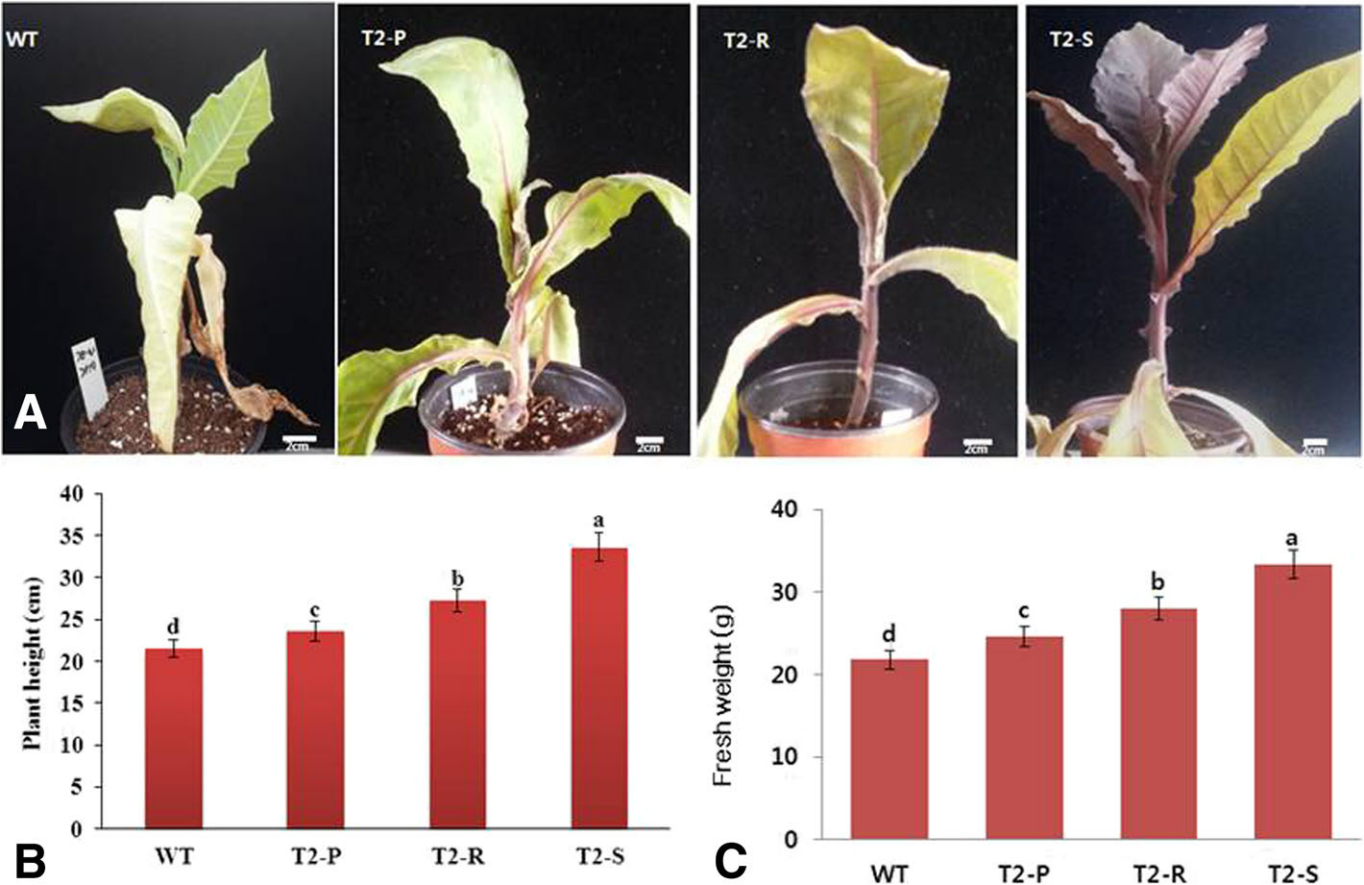
Comparisons of post-salt-stress (**a**) phenotypes, as well as (**b**, **c**) plant height and fresh weight in WT and transgenic *Del*-overexpressing plants (T_2_-P, T_2_-R, and T_2_-S). Error bars indicate the standard errors (SE) of average results

The effect of salt stress on both transgenic and WT plant growth was not obvious following irrigation with 50 mM NaCl (data not shown). However, increasing NaCl concentrations to 100 mM and 150 mM caused clear leaf curling and a decrease in shoot length. Under these conditions, WT leaves were succulent and brittle, while transgenic leaves appeared more normal (Fig. 6a). When NaCl concentration was increased to 200 mM and 250 mM, all plants exhibited obvious stunted growth, including reduced height. Under these conditions, T_2_-P and T_2_-R leaves drooped, while T_2_-S plants appeared more normal. A final increase of NaCl concentration to 300 mM severely affected all plant growth in the following order: WT > T_2_-P > T_2_-R > T_2_-S, as measured by impairments to plant height and fresh weight (Fig. 6b and c).

### Antioxidant activity in transgenic lines and WT after salt stress treatment

**Fig. 7:**
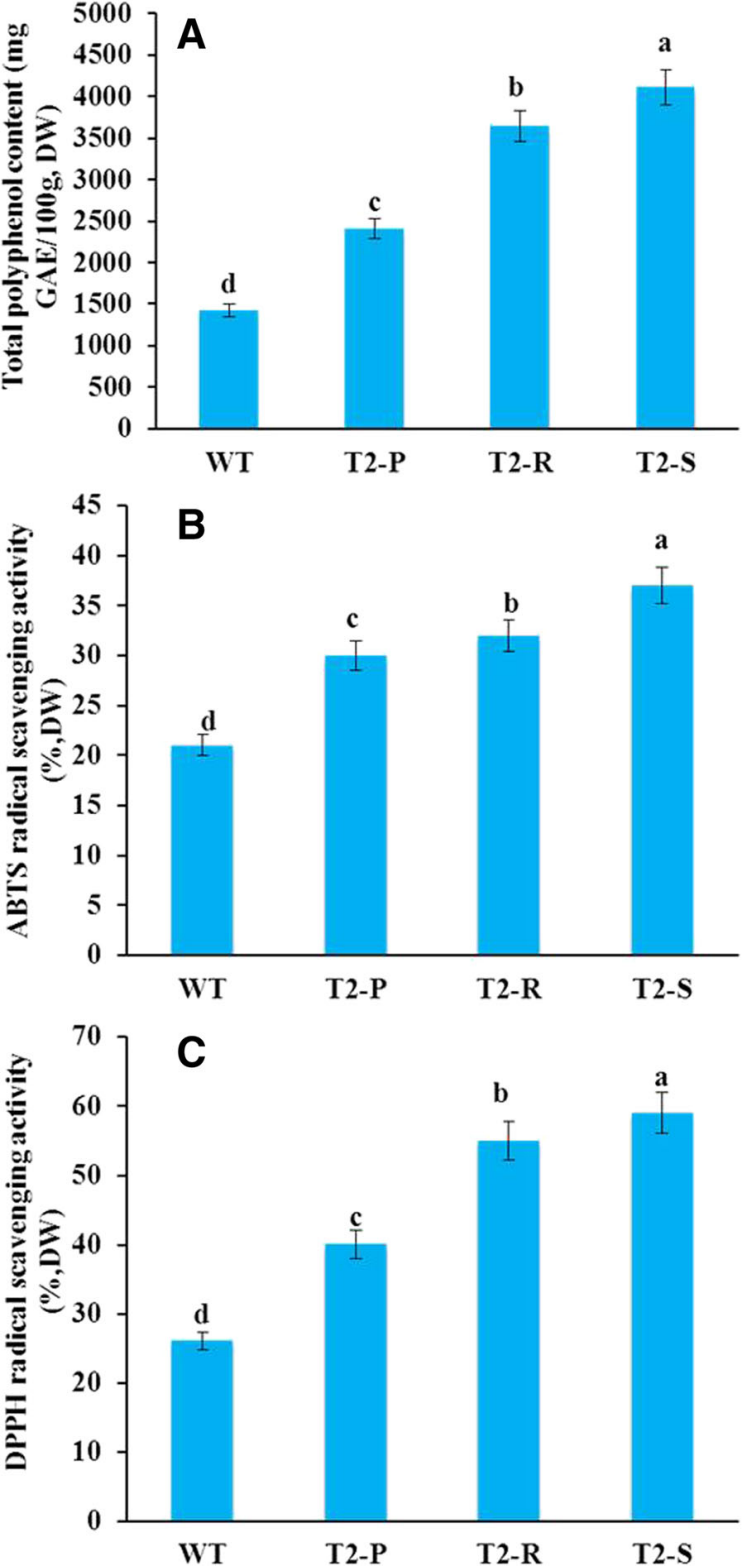
Comparisons of antioxidant activities post-salt-stress of WT and transgenic *Del*-overexpressing plants (T_2_-P, T_2_-R, and T_2_-S). (**a**) Total polyphenolic content, (**b**) ABTS radical scavenging activity, and **c**) DPPH radical scavenging activity. Error bars indicate the standard errors (SE) of average results

Following salt stress, antioxidant activity (ABTS and DPPH) and total polyphenol content of transgenic and WT plants were re-measured. Salt-stressed plants exhibited similar patterns in antioxidant activity and total polyphenol content as non-treated plants: highest in T_2_-S, intermediate in T_2_-R and T_2_-P, lowest in WT (Fig. 7a-c). However, absolute levels of both variables were distinctly lower in salt-stressed plants than in non-treated plants. In particular, differences between salt-stressed and non-treated WT plants were greater than those seen for the transgenic lines (Fig. 7a-c).

### Measurement of ionic compounds after salt-stress treatment

**Fig. 8:**
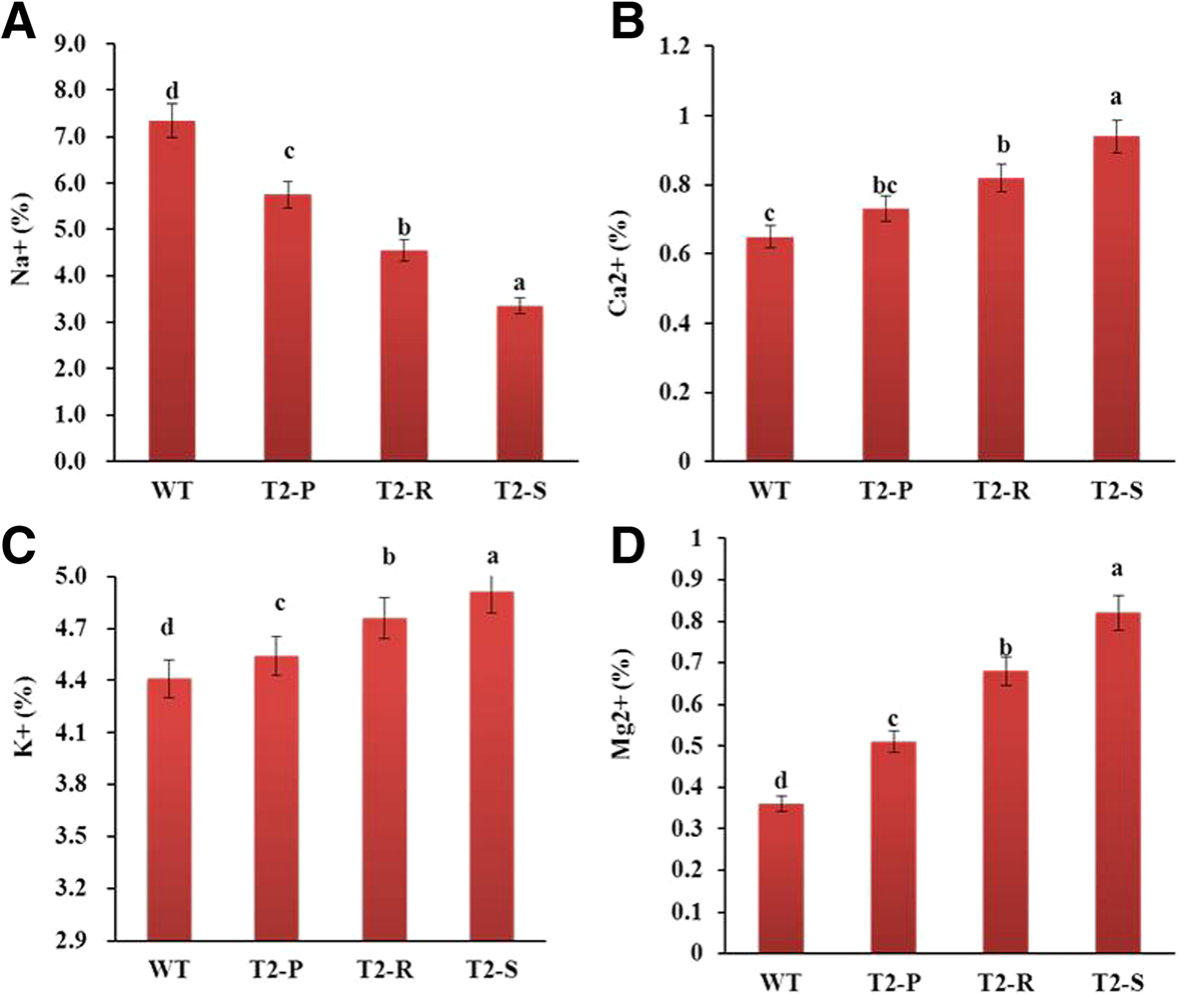
Ion content of leaf tissue following salt-stress treatment of WT and transgenic *Del*-overexpressing plants (T_2_-P, T_2_-R, and T_2_-S). (**a**) Na^+^, (**b**) Ca^2+^, (**c**) K^+^, and (**d**) Mg^2+^. Error bars indicate the standard errors (SE) of average results

The order of Na^+^ uptake was as follows: WT > T_2_-P > T_2_-R > T_2_-S (Fig. 8). Following salt-stress treatment, percentage accumulation of K^+^, Mg^2+^, and Ca^2+^ decreased in leaves, leading to the following order of ion content: WT < T_2_-P < T_2_-R < T_2_-S (Fig. 8).

### Effect of salt-stress treatment on gene expression

**Fig. 9:**
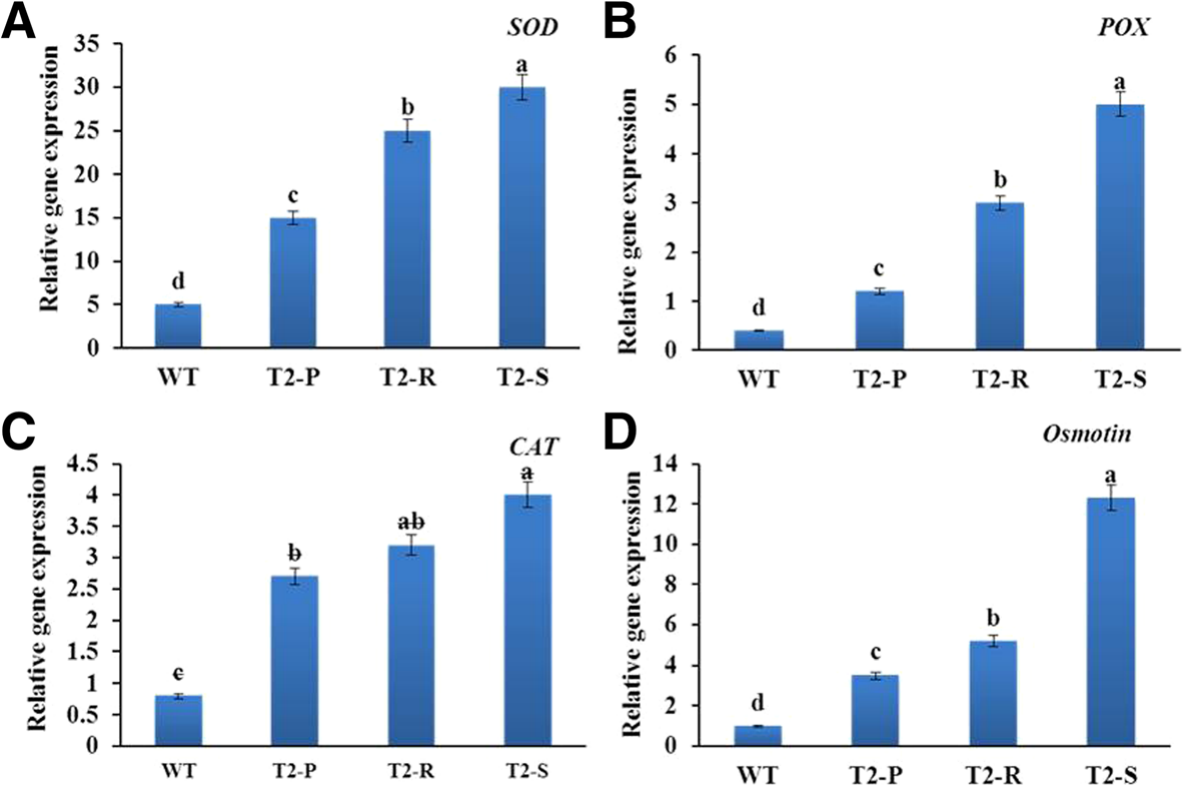
Analysis of antioxidant-related gene expression following salt stress of WT and transgenic *Del*-overexpressing plants (T_2_-P, T_2_-R, and T_2_-S). (**a**) *SOD*, (**b**) *POX*, (**c**) *CAT*, and (**d**) *Osmotin*. Error bars indicate the standard errors (SE) of average results

Transcript levels of *NtSOD*, *NtCAT*, *NtPOX,* and *NtOsmotin* were lowest in WT, then increased in transgenic lines as follows: T_2_-P < T_2_-R < T_2_-S (Fig. 9). Low gene expression in WT would lead to decreased production of antioxidant enzymes that provide salt-stress tolerance (i.e., SOD, CAT, and POX, and proline). Thus, this result supports the hypothesis that the higher salt-stress tolerance in transgenic lines depends on their antioxidant content.

### Drought stress tolerance

Under well-watered conditions, leaf size and plant height did not obviously differ between WT and transgenic plants. However, following 10 days of water deprivation, almost all WT plant leaves appeared wilted, whereas in transgenic plants, only lower leaves were wilted, while upper leaves remained green and fully expanded (data not shown). Thus, we evaluated physiological parameters linked to drought-stress tolerance, such as leaf stomatal density, relative water content (RWC), and MDA content.

**Fig. 10:**
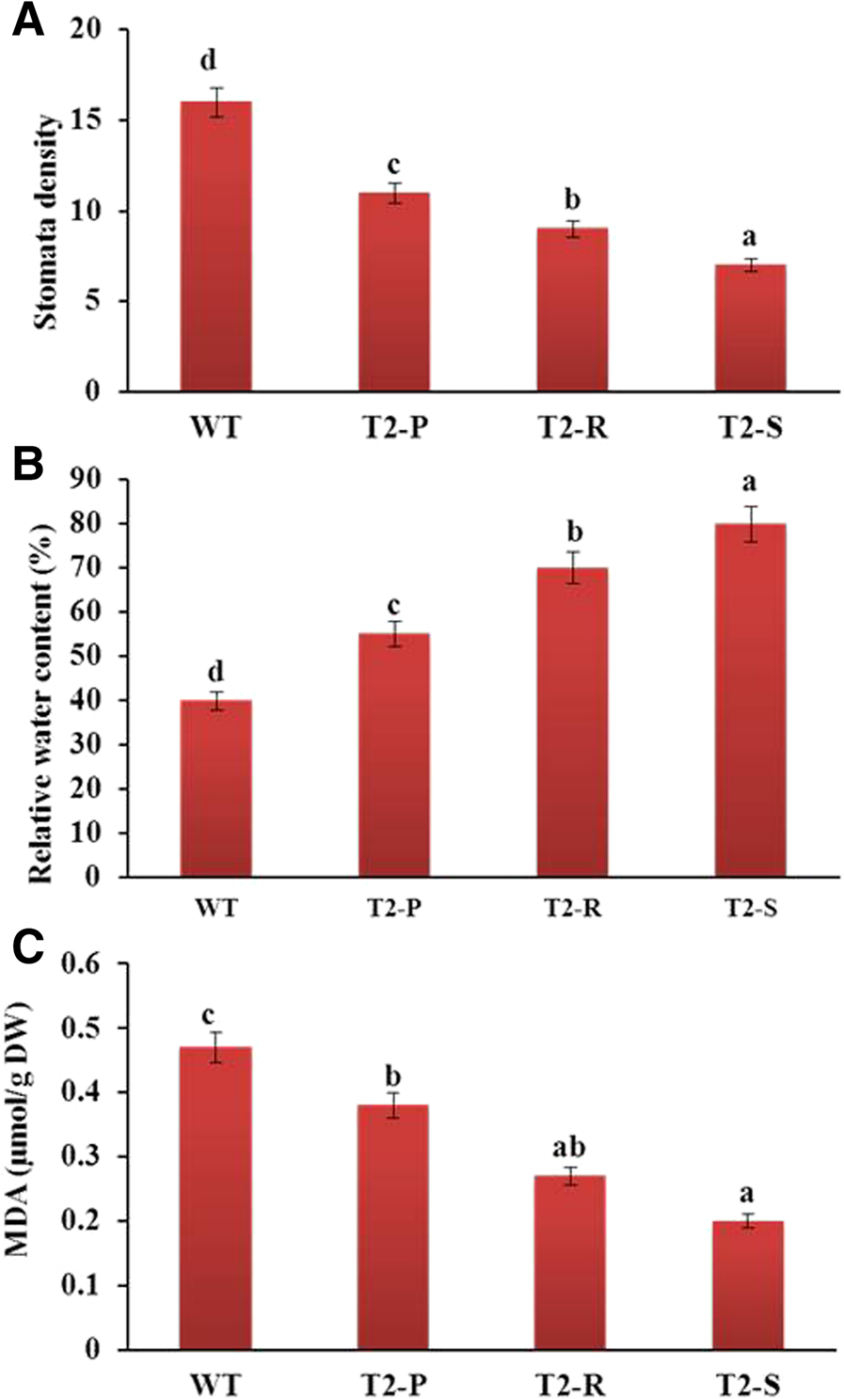
Comparison of leaf stomata density (**a**), relative water content (**b**), and MDA content (**c**) analyzed from the fifth top leaf of plants water-deprived for 10 days. Error bars indicate the standard errors (SE) of average results

In general, drought stress tolerance depends on transpiration rate, which is affected by stomatal density. Stomatal density was in the order of T_2_-S < T_2_-R < T_2_-P < WT, while RWC was 40%, 55%, 70%, and 80% WT, T_2_-P, T_2_-R, and T_2_-S plants, respectively (Fig. 10a). Consistent with these results, the water holding capacity during 10 days of drought stress was highest in T_2_-S, followed by T_2_-R, T_2_-P, and finally WT (Fig. 10b). High RWC in T_2_-S plants could be partially attributed to low stomatal density. Next, MDA content was lowest in T_2_-S, then in T_2_-R, T_2_-P, and lastly, WT (Fig. 10c), suggesting that reduced MDA from anthocyanin accumulation led to improved drought stress tolerance in the transgenic lines.

**Fig. 11:**
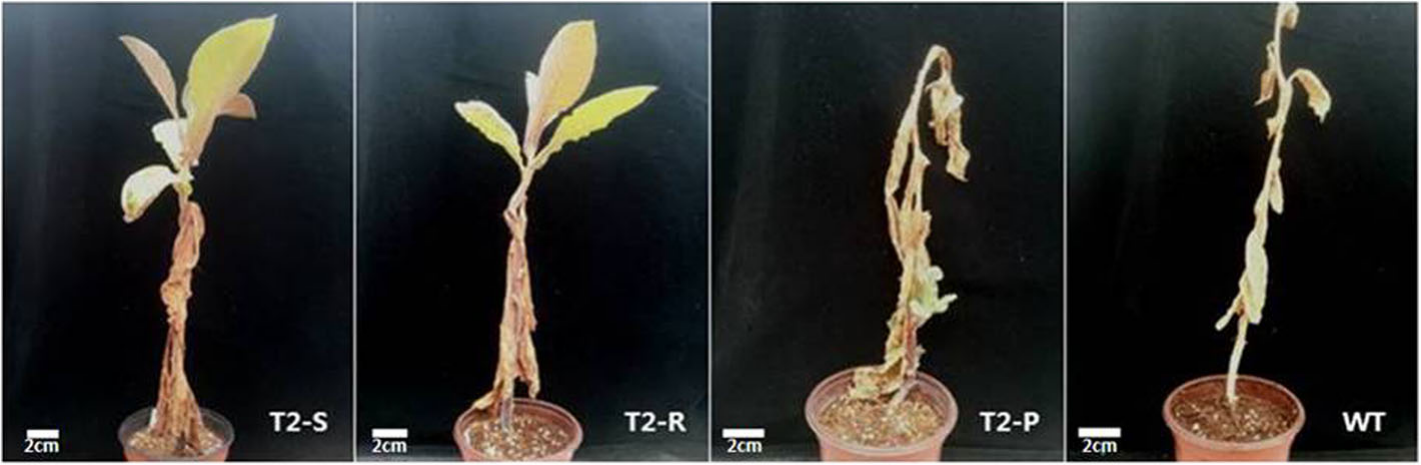
Comparison of plant recovery following drought stress (photos taken 3 days after re-watering) in WT and transgenic *Del*-overexpressing plants (T_2_-P, T_2_-R, and T_2_-S)

After 21 days of water deprivation, all WT and T_2_-P leaves turned yellow, with greater mortality in WT plants. Upper leaves of T_2_-R and T_2_-S plants displayed severe wilting only. After three days of re-watering, no WT plants recovered from drought stress, but new shoots from lower stem nodes were visible in T_2_-P plants (Fig. 11). Furthermore, T_2_-S and T_2_-R plants recovered well from drought stress, with their upper leaves comparable to those observed on well-watered plants (Fig. 11).

## Discussion

Although previous studies have examined the overexpression of regulatory genes affecting anthocyanins in various plant species, only a few reports have investigated the effect of *Del*. Thus, in this study, we focused on the involvement of *Del* in anthocyanin accumulation. In contrast to Mooney et al. [5], we found enhanced anthocyanin accumulation in vegetative tissue of transgenic *Del*-overexpressing tobacco. However, both the previous work and our current study observed enhanced anthocyanins in floral tissues (i.e., petal, corolla, and stamen). The between-study difference in anthocyanin accumulation of vegetative tissue is likely due to tobacco cultivar choice (‘Samsun’ in Mooney et al. [5]). Additionally, Mooney et al. [5] demonstrated that *NtCHS* and *NtDFR* absence explained the lack of anthocyanin accumulation in vegetative tissue. Our data support this conclusion, as we also did not detect *NtDFR* expression in the tobacco cultivar ‘Xanthi’ (WT) used here. Huang et al. [21] reported that *Lc* overexpression in tobacco upregulated *NtDFR* transcript levels, leading to purple pigmentation in young leaves at low temperatures, whereas in WT, *NtDFR* was not expressed and the pigmentation was absent. In this study, WT did not express either *NtDFR* or *NtANS*, while its expression of *NtCHS*, *NtCHI,* and *NtF3H* were relatively low. Thus, our results indicate that *Del* can regulate all biosynthetic genes and increase their expression levels. In addition, variation in anthocyanin accumulation among transgenic lines can be attributed to their differing biosynthetic gene transcript levels. However, some genes appear to play more important roles in enhancing anthocyanin production. As evidence, *NtANS* and *NtDFR* transcripts differed noticeably between T_2_-R and T_2_-P plants, whereas *NtCHS, NtCHI*, and *NtF3H* transcripts did not. Moreover, *NtDFR* transcripts were nearly identical between T_2_-R and T_2_-S plants, while higher *NtCHS*, *NtF3H*, and *NtANS* transcript levels were observed in the latter line. Collectively, these results indicate that *NtDFR* and *NtANS* are critical in anthocyanin production of T_2_-R and T_2_-P, while *NtCHS*, *NtF3H*, and *NtANS* are more relevant in T_2_-R and T_2_-S. Thus, although all biosynthetic genes are responsible for anthocyanin accumulation, *NtANS* was most crucial in transgenic lines. This finding distinguishes our study from previous reports [5] that did not investigate the role of biosynthetic genes in controlling differing anthocyanin accumulation levels. In another study [21], *NtDFR* absence was thought to be the major reason for anthocyanin biosynthesis inactivation in WT leaves, although *NtANS* transcript levels were distinctly higher than those of *NtDFR* in their transgenic lines. Nakatsuka et al. [22] also generated transgenic tobacco expressing 35S:*GhDFR* cloned from gerbera, but did not describe anthocyanin production in vegetative tissue. Two studies that did examine anthocyanins in vegetative tissues found VlmybA2 or B-peru + mPAP1 overexpression in tobacco to enhance anthocyanin accumulation in those plant parts [23, 24], but neither study investigated the role of biosynthetic genes in anthocyanin production.

In contrast to their expression in vegetative tissue, all anthocyanin biosynthetic genes were expressed in the pink WT flowers. Similarly, although *NtDFR* expression was absent in in the vegetative tissue of cultivar ‘Samsam’, it was detected in the light pink flowers [5]. Here, transgenic and WT flower colors mirrored vegetative tissue colors, with pigmentation increasing in the order of T_2_-S > T_2_-R > T_2_-P > WT. The qRT-PCR results indicate that these pigmentation differences are linked to variation in biosynthetic gene transcript levels, as the latter influences anthocyanin accumulation. Therefore, *NtCHI, NtDFR,* and *NtANS* are probably more critical for flower color differences in ‘Samsam’ due to their higher transcript levels. These results confirm the findings of Mooney et al. [5], who reported that *Del*-mediated upregulation of *NtDFR* expression in tobacco flowers enhanced pigmentation. In addition, Han et al. [25] also showed that inhibition of *NtCHI* and *NtDFR* expression in tobacco flowers decreased anthocyanins.

Through the overexpression of foreign biosynthetic and regulatory genes, Geekiyanage et al. [23] and Nakatsuka et al. [22] successfully modified tobacco flower color. However, they did not investigate how those transformed genes regulated native biosynthetic genes, nor did they examine how the biosynthetic genes influenced anthocyanin production. A similar study [24] also enhanced anthocyanin production in the same cultivar, but did not examine flower color patterns. Additionally, pigmentation patterns in young leaves of three *Lc*-expressing transgenic lines were investigated via examining anthocyanin biosynthetic gene transcript levels [21], but flower pigmentation patterns remained unexamined. Finally, a report demonstrating four new flower phenotypes from four independent, *35S-Tag1-R* transgenic lines did not evaluate those phenotypes with molecular characterization. Overall, *Del* overexpression in tobacco upregulates all anthocyanin biosynthetic genes, significantly increasing anthocyanin accumulation in vegetative and flower tissues. Thus, differences in anthocyanin accumulation across transgenic tobacco lines overexpressing *Del* are probably due to variation in anthocyanin biosynthetic gene transcript levels.

Environmental stresses that cause ROS generation are major threats affecting plant growth and yield. Owing to the threat of global climate change, plant scientists are increasingly interested in how plants sustain their yield under environmental stresses. According to the studies to date, when plants are exposed to environmental stress conditions, anthocyanin serves as an antioxidant enzyme and increases ROS scavenging activity to prevent plant damage [21, 26]. Oh et al. [27] suggested that enhanced anthocyanin content significantly increases *Arabidopsis* survival under salt stress. Similarly, anthocyanin increase confers heightened ROS scavenging activity in *Melissa officinalis* leaves [28]. Several other studies have also revealed that overexpression of anthocyanin regulatory gene *IbMYB1* in potato [17] and anthocyanin biosynthetic gene *CHS* in tobacco [29] enhances anthocyanin production and salt stress tolerance. Additionally, *MYB* overexpression in *Arabidopsis* plants causes anthocyanin over-accumulation, an outcome directly associated with oxidative- and drought-stress tolerance [20]. Moreover, overexpression of *Scutellaria baicalensis R2R3-MYB* genes (*SbMYB2*, *SbMYB7*, *SbMYB8*) regulates anthocyanin biosynthesis and improves abiotic stress tolerance in tobacco [30, 31]. Likewise, expression of a grape bHLH transcription factor gene (VvbHLH1) increases flavonoid accumulation, leading to enhanced salt and drought tolerance in transgenic *Arabidopsis* [32]. Although *Del* overexpression in tobacco and tomato enhances anthocyanin production [3, 33], only one study before this current report investigated the influence of *Del* on salt and drought stress tolerance (in transgenic Arabidopsis) [34]. Unlike our study, however, Wang et al. [34] did not find a link between *Del* overexpression and anthocyanin accumulation in transgenic *Arabidopsis*. Further, they did not document any correlation between anthocyanin content and degree of stress tolerance.

Depending on anthocyanin accumulation levels, ABTS and DPPH radical scavenging activities and total polyphenol content varied across transgenic lines, but were lowest in WT. This result indicates that high anthocyanin accumulation is likely to be associated with better ROS scavenging activity (antioxidant activities), supporting the findings of Lim et al. [35]. These authors also reported that *RsMYB1* overexpression in *Arabidopsis* greatly enhances anthocyanin production and improves antioxidant activity over WT, but they did not examine antioxidant activity in relation to varying amounts of anthocyanin accumulation.

Salt-stress treatments inhibited plant growth, lowered polyphenolic contents, and dampened antioxidant activities in every line. This outcome may be due to phenol and antioxidant consumption by plants to defend against salinity-induced oxidative stress. Throughout the stress treatment, stress tolerance was highest in T_2_-S, followed by T_2_-R, T_2_-P, and lastly, WT. This variation in stress tolerance seems due to inter-line variation in phenol content and antioxidant activity. As currently understood, high antioxidant activity is necessary to neutralize salt-stress-induced ROS, thereby preventing oxidative damage to proteins, lipids, and nucleic acids [36]. Similarly, polyphenols donate hydrogen atoms to neutralize ROS via creating water-soluble compounds [37], meaning high polyphenol content also increases stress tolerance.

Na^+^ uptake in WT was higher than in transgenic plants, and consequently, WT also contained lower concentrations of essential nutrients (e.g., K^+^, Ca^2+^, P^+^, and Mg^2+^). This deficiency in essential nutrients likely disturbed normal physiological function and is thus a factor inhibiting plant growth. Our results support the findings of Abdallah et al. [38], who demonstrated that high Na^+^ accumulation in *Solanum nigrum* affected essential nutrient uptake and reduced plant growth.

Antioxidant enzymes SOD, POX, and CAT are known to protect plants from oxidative damage during extreme stress conditions [39, 40]. Additionally, SOD, CAT, and POD actively scavenge ROS in salt-stressed plants [41, 42]. Notably, enhanced anthocyanin production increases SOD, CAT, and POD activities, and improves the survival rate of *Arabidopsis* seedlings under salt stress [42]. In this study, we found that salt-stress tolerance is likely linked to differential antioxidant gene expression in the transgenic lines and WT. Specifically, we observed that salt-stress tolerance (T_2_-S > T_2_-R > T_2_-P > WT) correlated well with *SOD*, *POX*, and *CAT* transcript levels. From the results, it seemed that overexpression of *Del* gene could enhance not only anthocyanin biosynthetic genes but also the antioxidant-related gene at transcript levels. Similarly, Wang et al. [34] also recently claimed that overexpression of *Del* gene could activate expression of the same genes. Many studies have also shown that overexpression of antioxidant genes heightens ROS scavenging activity and improves salt stress tolerance [43]. Thus, the possible explanation for the stress tolerance could be due to anthocyanin accumulation as well as induction of antioxidant-related genes.

Furthermore, Goel et al. [44] documented that *osmotin* overexpression in several plant species raises proline content, thereby increasing salt-stress tolerance. In this study, *osmotin* transcript levels also correlated well with salt tolerance (again, T_2_-S > T_2_-R > T_2_-P > WT). Hence, our work here corroborates previous studies showing a direct association between *osmotin* transcript level and degree of salt tolerance

Recent studies reported that anthocyanin accumulation was directly associated with drought tolerance in several plant species [19, 20, 31, 32, 34]. Under drought stress, transgenic plants generally exhibited better plant growth than WT. Here, we noticed that drought tolerance varied depending on anthocyanin accumulation (T_2_-S > T_2_-R > T_2_-P > WT), suggesting that the effect of *Del* overexpression in enhancing anthocyanin content and antioxidant activity also induces drought tolerance. Our conclusion is consistent with the findings of previous studies [19, 20, 31, 32, 34].

We observed here that anthocyanin-accumulating transgenic lines experienced a greater reduction in stomatal density than WT, consistent with previous studies [45, 46]. The recent studies that implicated anthocyanins in drought tolerance [19, 20, 31, 32, 34] did not examine any potential association between anthocyanin production and stomatal density reduction. Thus, our report is the first to link reduced stomatal density with anthocyanin production, providing a mechanism for increased drought tolerance in tobacco. On the other hand, reduction of the stomatal density could also be directly associated with overexpression of the bHLH transcription factor (*Del* gene) because overexpression of a bHLH transcription factor (PebHLH35) from *Populus euphratica* was found to confer drought tolerance by reducing stomatal density [47].

As an antioxidant, anthocyanins can scavenge ROS into vacuoles under drought stress, making them osmoregulators that maintain water homeostasis in plants [20]. Here, it is likely that anthocyanin accumulation prevented water loss in whole transgenic plants. Thus, our results support the findings of Nakabayashi et al. [20]. In addition, one possible explanation for less reduction of water in the plants could also be due to direct influence of the bHLH transcription factor (*Del* gene) because overexpression of the bHLH transcription factor (PebHLH35) delayed water loss in *Arabidopsis* [47].

Leaf MDA concentration is frequently used as an indicator of drought stress in plants, supported by data showing a direct association between low MDA concentrations and drought tolerance [34, 48]. In our work, MDA concentrations detected in *Del*-overexpressing transgenic lines had lower MDA concentrations than WT, and—as already stated—transgenic lines were also more resistant to water deficit. Thus, our data are in line with existing evidence showing that plants with lower MDA concentrations are more drought-tolerant. Recently, Wang et al. [34] also claimed that overexpression of *Del* gene enhanced flavonoid levels and lowered MDA content in *Arabidopsis*. Moreover, increased anthocyanin content in the transgenic lines were also found to be likely associated with lower MDA concentration.

## Conclusion

This study focuses on functional role of Snapdragon *Del* gene, a bHLH transcription factor, in anthocyanin production and abiotic stress tolerances for *Nicotiana tabaccum* cv. Xanthi. The results support that *Del* gene is able to enhance both anthocyanin production and the stress tolerance by regulating anthocyanin biosynthesis genes and antioxidant-related genes. Thus, we expect that *Del* gene could be exploited as a dual functional gene for improvement of anthocyanin production and the stress tolerances for horticultural crops.

## Methods

### Plant materials

Seven transgenic tobacco (cv. Xanthi, Japan Tobacco Co., Toyoda) lines were generated previously that expressed *Del* via the CaMV 35S promoter [33]. The 35S-*Del* gene construct was provided by Professor Cathie Martin (John Innes Centre, UK). Three lines among the T_0_ plants displayed different anthocyanin-accumulation phenotypes: pale red, red, and strong red; these lines were selected and labeled as T_0_-P, T_0_-R, and T_0_-S, respectively (Fig. 1a).

### Generation of T_2_ lines

To generate T_1_ plants, T_0_-P, T_0_-R, and T_0_-S plants were grown in a greenhouse (Fig. 1b) until flowering, and then self-pollinated. The resulting progenies were selected based on anthocyanin accumulation phenotype. Similarly, the final T_2_-P, T_2_-R, and T_2_-S plants used for further analysis were obtained through self-pollination of T_1_ plants, then subjected to the same phenotype screening process for anthocyanin accumulation.

### Analysis of total anthocyanin content

Ten plants from the T_2_ generation per line were selected for analysis of total anthocyanin content, following Ai et al. [49]. Briefly, ~500 mg) of leaves per plant was grounded to a fine powder, which was transferred to an extraction solution for obtaining anthocyanins. The extract was incubated at 4 °C for 24 h, then centrifuged at 13 000 rpm and 4 °C for 20 min. The supernatant containing anthocyanin was analyzed with a spectrophotometer (Shimadzu, Kyoto, Japan). Error bars indicate the standard error (SE) of average anthocyanin content.

### RNA extraction and RT-PCR analysis

To determine anthocyanin biosynthetic gene expression in T_2_-P, T_2_-R, and T_2_-S plants, total RNA was isolated from 100 mg of leaf tissue per line (transgenic + WT) using TRI Reagent^TM^ Solution (Ambion, USA). The synthesis of cDNA was performed using Transcription Kit (Applied Biosystems, USA). Table 1 lists the primers and PCR conditions for amplification of *NtCHI, NtCHS, NtF3H, NtDFR,* and *NtANS*, as well as the reference *ACTIN* gene.

**Table 1:** Primer sequences for RT-PCR and qRT-PCR of anthocyanin biosynthetic genes

Gene	Accession No.	Primer sequences
*CHS*	AF311783	F, 5′- TTGTTCGAGCTTGTCTCTGC −3′
		R, 5′- AGCCCAGGAACATCTTTGAG-3′
*CHI*	AB213651	F, 5′- GTCAGGCCATTGAAAAGCTC −3′
		R, 5′- CTAATCGTCAATGCCCCAAC −3′
*F3H*	AB289450	F, 5′- CAAGGCATGTGTGGATATGG-3′
		R, 5′- TGTGTCGTTTCAGTCCAAGG −3′
*DFR*	AB289448	F, 5′- AACCAACAGTCAGGGGAATG −3′
		R, 5′- TTGGACATCGACAGTTCCAG-3′
*ANS*	AB289447	F, 5′- TGGCGTTGAAGCTCATACTG −3′
		R, 5′- GGAATTAGGCACACACTTTGC −3′
*ACTIN*	AB158612	F, 5′- GGTCATTACCATTGGCTCAGA −3′
		R, 5′- CAACAAGTGATGGCTGGAATAAA −3′

*CHS* chalcone synthase, *CHI* chalcone isomerase, *F3H* flavanone-3-hydroxylase, *DFR* dihydroflavonol 4-reductase, *ANS* anthocyanidin synthase, *ACTIN* reference gene used for expression normalization

PCR conditions for RT-PCR: 95 °C (2 min) → [95 °C (20 s) → 57 °C (40 s) → 72 °C (30 s)] × 35 cycles → 72 °C (5 min) → 4 °C

PCR conditions for qRT-PCR: 95 °C (10 min) → [95 °C (30 s) → 57 °C (25 s) → 72 °C (35 s)] × 40 cycles → 95 °C (15 s) → 57 °C (30 s) → 95 °C (15 s)

### Transcriptional analysis of anthocyanin biosynthetic genes in vegetative and floral tissues using qRT-PCR

Total RNA isolated from leaf and floral tissues of T_2_ plants were analyzed. Following manufacturer protocol, 1 μg of total RNA and an oligo dT_20_ primer were used for reverse transcription (ReverTra Ace-á, Toyobo, Japan). Transcript levels of *NtCHI, NtCHS, NtF3H, NtDFR, NtANS,* and *ACTIN* were measured using a StepOnePlus Real-Time PCR system (Thermo Fisher Scientific, Waltham, USA) (Ai et al. 2016). The primers and PCR conditions for the detected genes are listed in Table 1. Five samples per line were used, and the analysis was repeated three times.

## Environmental stress experiments

### Plant materials

Seeds of T_2_-P, T_2_-R, T_2_-R, and WT tobacco plants were germinated on separate seedling beds in a greenhouse with an average temperature of 25 °C. Seedlings were allowed to grow for three weeks. Subsequently, uniform, healthy WT and transgenic plants with the appropriate anthocyanin phenotypes were transferred to individual pots. Prior to performing stress treatments, DPPH and ABTS radical scavenging activity, as well as total polyphenol content, were determined for each line (5 plants per line) following the methods of Kim et al. [50] and Lim et al. [35].

### Salt stress

Three-week-old T_2_-P, T_2_-R, T_2_-S, and WT plants (20 per line) were grown for another week under normal conditions before being subjected to an irrigation treatment every six days with increasing NaCl concentrations (50, 100, 150, 200, 250, and 300 mM). Growth variables (plant height and fresh weight) were evaluated at the end of irrigation, and the experiment was repeated three times. Plants were maintained in a growth chamber set to 22 °C and ~60% relative humidity with a 16-h photoperiod.

### Radical scavenging activity assay

Radical scavenging activity from five samples per line was measured before and after salt stress using DPPH and ABTS assays, repeated in triplicate, (following Kim et al. [50]; Lim et al. [35]).

### Determination of total polyphenol content

Total polyphenolic content per line was determined before and after salt stress, following Dewanto et al. [51], five samples per line were used, and the analysis was repeated three times.

### Determination of major ion content

Post-salt-stress treatment, Na^+^, K^+^, Ca^2+^, and Mg^2+^ were extracted from 1 g of dried leaf tissue per line using 0.5% HNO_3_. Ion content was then assayed through ion conductivity, as described by Cataldi et al. [52]. Measurements were performed in five samples with three replicates.

### RNA extraction and analysis of gene expression by qRT-PCR

To determine *NtSOD*, *NtCAT*, *NtPOX*, and *NtOsmotin* expression levels, qRT-PCR analysis was performed. Leaf tissue per line was sampled following salt-stress treatment. Total RNA extraction and analysis of gene expression by qRT-PCR were performed as described under “RNA extraction and RT-PCR analysis,” as well as “Transcriptional analysis of anthocyanin biosynthetic genes in vegetative and floral tissues using qRT-PCR.” *ACTIN* was used as an internal reference gene. Tables 2 and 3 list the primers and PCR conditions, respectively. Analysis was performed in five samples with three replicates.

**Table 2:** Primer sequences for qRT-PCR of antioxidant- and proline-related genes

Gene	Accession No.	Primer sequences
*SOD*	EU342358	F; 5′- GCCAGCTTTGAAGATGAACGA-3′
		R; 5′- GCCTAATGCTCTTCCCACCAT-3′
*CAT*	U93244	F; 5′- GATGACAAGATGCTTCAAACTCGTA-3′
		R; 5′- CACTTTGGAGCATTAGCAGGAA-3′
*POX*	D11396.1	F; 5′- ACTGCTCCGTCACCCAAAAC-3′
		R; 5′- GCCCTGGTTCCTGCTTAAGTC-3′
*Osmotin*	X95308	F; 5′- ACTATCGAGGTCCGAAACAACTG-3′
		R; 5′- GCATTGATCACCCAAGTTTGG-3′

**Table 3:** PCR conditions for qRT-PCR of antioxidant- and proline-related genes

Genes	PCR conditions:
*SOD*	95 °C (10 min) → [95 °C (30 s) → 59 °C (30 s)] × 40 cycles → 95 °C (15 s) → 59 °C (30 s) → 95 °C (15 s)
*CAT*	95 °C (10 min) → [95 °C (30 s) → 59 °C (30 s)] × 40 cycles → 95 °C (15 s) → 59 °C (30 s) → 95 °C (15 s)
*POX*	95 °C (10 min) → [95 °C (30 s) → 60 °C (30 s)] × 40 cycles → 95 °C (15 s) → 60 °C (30 s) → 95 °C (15 s)
*Osmotin*	95 °C (10 min) → [95 °C (30 s) → 59 °C (30 s)] × 40 cycles → 95 °C (15 s) → 59 °C (30 s) → 95 °C (15 s)

### Drought stress

Three-week-old T_2_-P, T_2_-R, T_2_-S, and WT plants (20 per line) were grown for another week under well-watered conditions before a three-week water deprivation. Subsequently, normal watering conditions were reinstated for 3 days. Growth responses of the plants to drought stress and re-watering were evaluated, and the experiment was repeated three times.

### Determination of relative water content (RWC)

Post-drought-stress RWC was determined using the fifth leaf from the top of plants subjected to 10 days of water deprivation. Leaves were excised and fresh leaf weight was immediately recorded. Leaves were then floated in deionized water at 4 °C overnight before recording their rehydrated weights. Finally, leaves were oven-dried at 70 °C overnight, and dry leaf weight was recorded. The formula for determining RWC is as follows: RWC = (fresh weight - dry weight) / (rehydrated weight - dry weight). Five leaves per line were used to determine RWC, and the analysis was repeated three times.

### Analysis of stomatal density

Leaf stomatal density was analyzed in the fifth leaf from the top of plants water-deprived for 10 days. Stomatal density was counted following method of Chung et al. [53]. Five leaves per line were used, and the analysis was repeated three times..

### Determination of malondialdehyde (MDA) content

Malondialdehyde content was determined in the fifth leaf from the top of plants water-deprived for 10 days, following method of Bates et al. [54]. Five leaves per line were used, and the analysis was repeated three times.

### Statistical analysis

Data were statistically analyzed in SPSS version 11.09 (IBM Corporation, Armonk, USA) and are presented as means ± standard errors (SE). Duncan’s multiple range test (DMRT) was used to separate the means, and significance was set at P < 0.05.

## Abbreviations

ABTS: 2, 2′-azino-bis-3-ethylbenzthiazoline-6-sulphonic acid
ANS: Anthocyanidin synthase
APX: Ascorbate peroxidase
bHLH: Basic helix-loop-helix
Ca^2+^: Calcium ion
CAT: Catalase
CHI: Chalcone isomerase
CHS: Chalcone synthase
*Del*: *Delila*
DFR: Dihydroflavonol 4-Reductase
DPPH: 1, 1-diphenyl-2-picrylhydrazyl radical
F3H: Flavanone-3-Hydroxylase
K^+^: Potassium ion
MDA: Malondialdehyde
Mg^2+^: Magnesium ion
Na^+^: Sodium ion
POX: Peroxidase
qRT-PCR: Quantitative real time polymerase chain reaction
ROS: Reactive oxygen species
RWC: Relative water content
SOD: Superoxide dismutase.
